# Adding Sound Transparency to a Spacesuit: Effect on Cognitive Performance in Females

**DOI:** 10.1109/OJEMB.2023.3288740

**Published:** 2023-06-22

**Authors:** Jose Berengueres, Mariam AlKuwaiti, Mohamed Abduljabbar, Fatma Taher

**Affiliations:** ^1^ College of ITUAE University 11239 Al Ain 17551 UAE; ^2^ Zayed University 54483 Dubai 144534 UAE

**Keywords:** Human factors, mars, sensory deprivation, sound, spacesuit

## Abstract

Spacesuits may block external sound. This induces sensory deprivation; a side effect is lower cognitive performance. This can increase the risk of an accident. This undesirable effect can be mitigated by designing suits with sound transparency. If the atmosphere is available, as on Mars, sound transparency can be realized by augmenting and processing external sounds. If no atmosphere is available, such as on the Moon, then an Earth-like sound can be re-created via generative AR techniques. We measure the effect of adding sound transparency in an Intra-Vehicular Activity suit by means of the Koh Block test. The results indicate that participants complete the test more quickly when wearing a suit with sound transparency.

## I. Introduction

Intra-Vehicular Activity (IVA) and Extra-Vehicular Activity (EVA) safety is susceptible to numerous factors, with astronaut fatigue and cognitive load being primary concerns, often closely interlinked. Astronaut fatigue chiefly stems from the considerable weight (>100 kg), pressurization, and restricted mobility of spacesuits [1]. High cognitive load arises from the abundance of error-sensitive tasks, compounded by sensory deprivation [2], [3]. EVA spacesuits, comprised of up to 16 material layers, impose limitations on touch, and visibility (e.g., 2023 Artemis spacesuits obstruct foot view, and block sound [4]). The consequence of this degradation of senses is an increase in cognitive load [5], [6] and the effect on mission safety can be as critical as those of fatigue. This effect was evidenced in a 2021 analysis that shows a correlation between cognitive load and increased falls during the Apollo missions [7].

### A. Ergonomics and Safety

Astronauts suffer a surprisingly high rate of musculoskeletal injury [8]. Various authors have addressed the ergonomics of suits from different perspectives [9], [10], [11]. Further efforts beyond ergonomics have been proposed in diverse areas, such as hypercapnia prevention, thermal regulation, waste management, radiation shielding, and further injury prevention [12], [6], [13]. Solutions to mitigate sensory degradation have also been proposed via the use of various interfaces, such as multimodal screens [14], Augmented Reality [15], new glove designs [16], and passive electro-stimulation for haptic sensory substitution [17], among others. Compared to other solutions such as haptics [18], sound design [19] has been an overlooked area that offers potential for improvement with minimal engineering drawbacks.

### B. Auditive Comfort

EVA and IVA suits offer life support [21], [22] but tend to dampen external sounds, especially those associated with EVA activities. Consequently, the protective features of these suits limit the natural auditory experience that humans typically encounter in their daily environment. The suits often incorporate multiple layers of insulating and protective materials, which can attenuate sound transmission and lead to muffled or reduced audio clarity. For example, when wearing Final Frontier Design's training suit with visor down (Fig. 2), students reported an inability to understand speech. Consequently, individuals wearing an IVA suit might face challenges in perceiving and interpreting auditory information, which could have a substantial impact on their cognitive performance and problem-solving abilities in situations where sound affects cognition [3], [7].

**Fig. 1.**
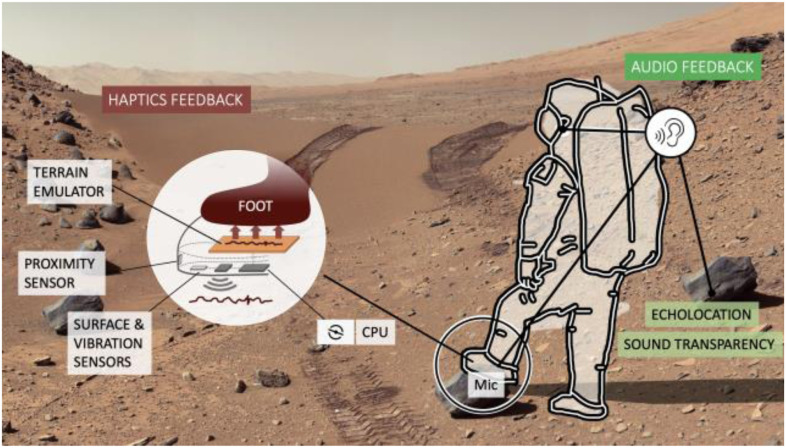
An enhanced EVA suit concept. Efforts to mitigate sensory deprivation in suits have been focused on haptics (left) while auditive comfort has been largely overlooked (right). Background image: Mast Camera (Mastcam), Curiosity's (9 February 2014), NASA/Caltech.

**Fig. 2.**
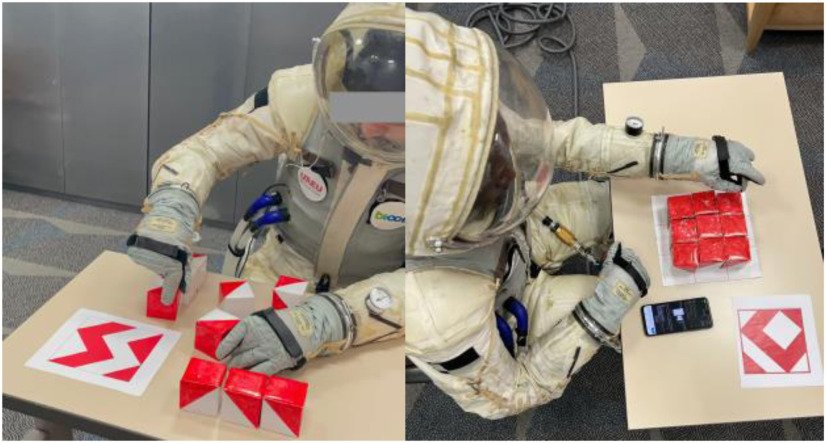
Two participants undertake their respective Koh Block tests. Left: test is mid journey, sound transparency OFF. Right: start of the test. Note the smartphone placed on the table used to relay ambient sound to the participant (sound transparency ON).

In the case of an EVA occurring in the absence of atmosphere, the sound and vibrations that the astronaut feels are transmitted by the suit itself from the suit's contact with objects, or contact and friction between the human skin and the suit's inner shell. Therefore, the absence of atmosphere does not imply silence, as the suit (that is analogous to a pressurized human-sized balloon) transmits sound and vibrations to its wearer. In addition, on a hypothetical Mars walk the rarified Martian atmosphere would provide faint audio feedback, which has a significantly different signature from feedback heard on Earth. Regardless, of the fact that on Mars the suit would block external sounds too, it is not unfeasible to process the Martian sounds to make them sound like they would on Earth by means of simple sound processing techniques and the use of linear filtering techniques.

### C. Evaluation of Sound Transparency

Our primary objective is to assess the impact of sound transparency on cognitive performance in subjects wearing an IVA spacesuit. We hypothesize that the implementation of transparency leads to enhanced cognitive performance in tasks that require manipulation as well as problem solving skills. To test this hypothesis, we designed an experimental setup with two conditions: a standard spacesuit and one where an audio system provides sound transparency. We measure cognitive performance using the Koh block test.

The Koh Block test is about solving a 9-block puzzle. It requires manual dexterity as well as problems solving skills. It was chosen because it is representative of the task an astronaut faces, such as during a spacewalk (assembly, tool manipulation, thinking, stress, and time constraints). We considered various tests. In our related work [20], we applied the Fukuda step test to measure cognitive improvement related to proprioception in preventing falls during EVA; however, the results were inconclusive as the Fukuda test is primarily designed to detect vestibular damage. Another alternative is to use the NASA Task Load Index; although widely used in various fields [21], including to assess sound comfort in offices [22], it is not a well-defined test per se but rather a qualitative assessment guide that can be applied to any activity. Thus, we opted for the Koh Block Test, which is quantitative. As it is timed, it facilitates a numerical comparison between different suit configurations (sound transparency ON/OFF).

## II. Results

**Fig. 3.**
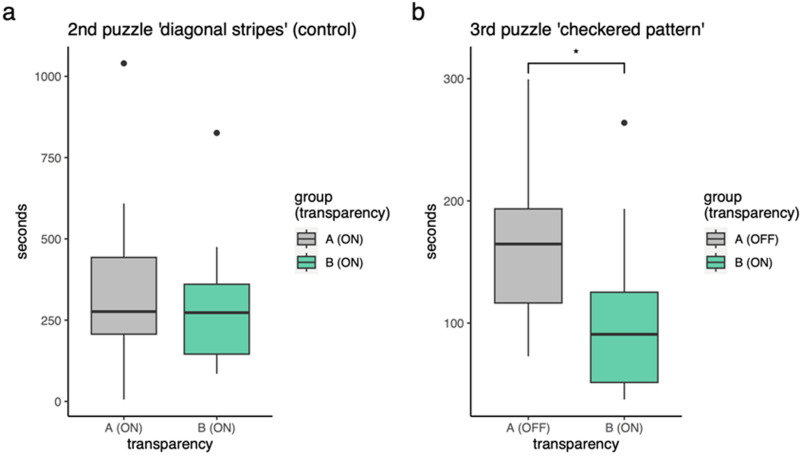
The time taken to complete a puzzle. (a) A control experiment where both Group A and B wear a Terra-Suit with sound transparency ON. (b) group A wears the Terra-Suit suit without sound transparency (OFF), group B wears the Terra-Suit with sound transparency ON.

We divided the participants into two groups. Fig. 3(a) compares groups' puzzle completion times using sound transparency ON for both. Fig. 3(b) compares puzzle completion times when sound transparency is ON for one group and OFF for the other, statistical significance (p<0.05) is indicated with an asterisk. See also the sequence section in the materials and methods.

### A. Effect of Sound Transparency on Competition Time

Welch's t-test shows a significant difference (t = 2.38, p = 0.012) in completion time between the groups with sound transparency ON (mean = 101 s) and OFF (mean = 159 s), with a 95% CI (16.6 s, Inf). The results support the argument that providing astronauts with sound transparent suits improves cognitive performance. A summary of qualitative comments from participants is shown in Table I. It shows a list of the most common feedback from participants aggregated by topic.

**TABLE 1:** Top 5 Topics in Participant Feedback

**Aggregated topic**	**Participants**
Hard to grip and move hand	23
Sound transparency was helpful	17
First Koh puzzle was the hardest	8
Second Koh puzzle was the hardest	6
Third Koh puzzle was the easiest	6

## III. Discussion

Of the more than 500 astronauts that have flown to space, about 11% have been women. The median age was 46 years old.

### A. The Gender Gap in Space

In the past astronauts used to belong to a very specific demographic of race and gender. While this distribution has changed in recent missions with improvements in gender [23] and race distributions, society, however, is still far away from gender equality. This research pioneers a gendered approach because it focused on a sample of 95% females that were younger than the average astronaut. This approach manifest due to our own logistic limitations.

### B. Generalization of Results

#### 1) Gender

The literature related to our setup does not support that comparative the results would be dissimilar in the case of the male-only population [24]. However, more study might be needed to account for consequences of long exposure to the space environment.

#### 2) Age

The average age of the sample here is 22 years old. The youngest astronaut case was the orbital flight of Gherman Titov in 1961 who was 25 at the time. We acknowledge that the results may not be generalizable across different age groups. Age can have a significant impact on cognitive performance, with research suggesting changes in cognitive abilities, such as processing speed, working memory, and problem-solving, over the course of one's lifetime [25], [26]. Future work could investigate the effects of age on problem-solving efficiency and compare the results with our findings.

#### 3) Grade Point Average

Finally, we observed no correlation (R < 2%) between self-declared Grade Point Average (a numerical score of academic performance) and time to complete the test. This finding is in line with current literature. While some studies have identified a correlation between Intelligence Quotient and academic outcomes, they also highlighted the importance of other factors, such as self-discipline, having a greater influence [27]. Further studies concluded that Intelligence Quotient alone does not account for the differences in academic performance among students, with motivation being the primary factor [28].

## IV. Conclusion

This study highlights the importance of sound transparency in spacesuit audio systems, demonstrating its impact on cognitive performance. Our findings demonstrate that the implementation of transparency improves cognitive performance, with potential implications for spacesuit design, safety. Other areas of application of these findings are underwater welding operations as they have similar sensory impairments and record one of the highest occupational fatality rates in the world [29].

## V. Materials and Methods

### A. Experimental Setup

An IVA training suit (Terra-Suit) from Final Frontier Design [30], [31] (NY) was rented from startup Zero2infinity S.A, Barcelona, Spain, and connected to a Stanley FATMAX air compressor (30 L/min, 59 dB noise). A digital air regulator (LEMATEC DAR02B) maintained a constant pressure inside the suit between 1.01–1.03 bars. Test days had temperatures of 22–24 °C, and the suit had a sound insulating characteristic of 21 dB. Apple AirPods^TM^ (1st generation) paired with a microphone Android App was used to provide sound transparency, with a one-way (mic-2-ear) delay of 144 ms. The experiments were carried in room E1-3071 in the campus of the first author. This room was windowless, and had a relatively high background noise level of 42.5 dB stemming HVAC, which was always on. During the experiments, the noise level was 49.5 dB, owing to the compressor that sat 3 m away. The compressor was always on. Flooring: carpet flooring. Lighting: fluorescent bulbs. Table used: standard 1m by 40 cm. Chair, standard issue wood and steel chair. See Fig. 2

### B. Koh Block Test

A description of the Koh block protocol can be found in [24]. Procedure: Participants were briefed, given consent forms, and informed of the procedure. They were instructed to solve **three** different Koh block puzzles in sequence: (Figs. 2-3). All participants solved the puzzles in the same order, with an approximate duration of 23 minutes per participant.

#### 1) Sequence

*Briefing and consent form signing.*

***First*** *puzzle: Participants familiarized themselves with the Koh block test mechanics by solving the ‘offset diamond’ puzzle without wearing the suit*.

*Spacesuit-donning, EarPods that relay exterior sound via Bluetooth are placed on participants’ ears*.

*Visor down.*

***Second*** *puzzle (control experiment): Both groups A and B, solve the ‘diagonal stripes’ puzzle. Sound transparency is on*.

*Visor up.*

*EarPods removed from Group A participants. Feedback is not requested but noted down if any is received spontaneously*.

*Visor down.*

***Third*** *puzzle: Both groups A and B solve the ‘checkered pattern’ puzzle*.

*Visor up.*

*Qualitative feedback is requested and collected. Removal of the suit*.

#### 2) Participant Demographic

39 participants were recruited from UAE University (36 female, 3 male), with a mean age of 21.5 (SD=4.6, min 18, max 33). Three participants were left-handed, six wore contact lenses or prescription glasses, and various preexisting conditions were reported. Participants signaled puzzle completion with a hand gesture. Time was recorded, and the experiment ended. If a participant completed the puzzle incorrectly, their data is not used, which occurred for seven participants across the three puzzles. Remaining statistics for groups A and B: Group A (18 females, mean age 20.4, five used graduated glasses); Group B (16 females, 2 males, mean age 20.0, two used graduated glasses). Male performance was similar or worse to mean female performance.

### C. Data Availability

The Data set is available at IEEEDataPort https://dx.doi.org/10.21227/12jp-pq48

### D. Ethics

This research, titled "Space Suit Haptics Project," adhered to IEEE and UAEU ethical guidelines. The study received ethical approval from the UAEU Social Sciences Ethics Committee - Research / Course (Application No: ERSC_2023_2408) on January 20, 2023. Key ethical considerations were addressed as follows:

1. Informed Consent: Participants provided written consent after receiving a detailed explanation of the project.
2. Privacy and Confidentiality: Participant data was securely stored and anonymized to maintain privacy.
3. Minimization of Potential Harm: Precautions were taken to avoid undue physical, psychological, or emotional risks to participants.
4. Fair Treatment: All participants were treated fairly and without discrimination during recruitment and throughout the study.
5. Transparency and Accountability: The research team disclosed methods, findings, and potential conflicts of interest.
